# The trust game: CRISPR for human germline editing unsettles scientists and society

**DOI:** 10.15252/embr.201847583

**Published:** 2019-01-02

**Authors:** Matthias Braun, Darian Meacham

**Affiliations:** ^1^ Friedrich‐Alexander‐University Erlangen‐Nuremberg Erlangen Germany; ^2^ Maastricht University Maastricht The Netherlands

**Keywords:** Genetics, Gene Therapy & Genetic Disease, S&S: Ethics, S&S: Politics, Policy & Law

## Abstract

The reaction to the alleged birth of two genome‐edited babies in China reveals deeper problems with the oversight of gene editing technologies and public trust in its applications.

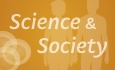

In November 2018, the Chinese researcher Jiankui He claimed that he and his colleagues had gene‐edited human embryos, resulting in the birth of twins with a modified genome. His announcement was met with near universal denunciation by leading scientists, ethicists, and policymakers from all over the world, including his home institution, the Southern University of Science and Technology in Shenzhen. Notwithstanding some nuances, they all condemned He's work on scientific and ethical grounds. A total of 122 Chinese scientists issued an open statement that “this presents a major blow to the image and development of Chinese life sciences on the global stage […] and is extremely unfair for the many honest and sincere scholars working to adhere to moral practices in the sciences” (http://3g.163.com/news/article_cambrian/E1IBVFD20001899O.html). The Chinese vice minister for science and technology also weighed in: “The genetically edited infant incident reported by media blatantly violated China's relevant laws and regulations. It has also violated the ethical bottom line that the academic community adheres to. It is shocking and unacceptable” (http://m.news.cctv.com/2018/11/29/ARTIqnwNDtHx6ThPOqqWDmzI181129.shtml). As a result, the Chinese government pledged to launch a criminal investigation. In a nutshell, a Chinese researcher who was ensnared by the promise of ground‐breaking work made an inexcusable mistake. Across the board, a central message was that He's work was a breach of trust—as for example George Church argues 1.

## Just individual misconduct?

But this is only one part of the picture. While this scientific misconduct may still be dealt with within the realm of science, there are two more conceptual problems. The first is the question of who should and could decide on how a technology is used, for what purpose, and with which societal consent. While the birth of gene‐edited children may be a rogue action, it reveals that there is insufficient oversight of the use of CRISPR or other gene‐editing technologies, despite international efforts to find a common position. There is no international framework to monitor this research as exists, for example, with the Biological Weapons Convention to prevent the development of biological weapons, or laws and regulation about the release of genetically modified organisms into the environment. Such a framework is not only necessary in order to define acceptable goals of research and development, but, more fundamentally, important in helping to establish a consensus of public knowledge concerning scientific evidence. Thus, the story of He and his colleagues may be interpreted as an urgent plea for finding a common ground on which to discuss and weigh scientific facts and evidence regarding manipulations of the human germline.

The fact that He announced the birth of CRISPR babies a few days before the Second International Summit on Genome Editing in Hong Kong is a reminder that there have already been negotiations within science as well as between science and society. The starting point was the publication of CRISPR/Cas experiments on non‐viable human embryos that explored the prospect of human germline modification 2. This prompted a call for a moratorium by scientists and the first International Summit on Human Gene Editing in Washington, D.C. in December 2015. This summit concluded with the need to “reach a broad societal consensus about the appropriateness of the proposed application” of CRISPR 3. The consensus—backed by the Oviedo Convention as well as nearly all scientific and political institutions—seemed to be that gene‐editing technologies should be used for therapeutic applications, but not for manipulating the human germline in the context of reproduction.

## Shifting perspectives

But this agreement has come under pressure, in large part because of further research that demonstrated the feasibility of correcting mutations in human embryos 4 as well as institutional statements that seem to soften the stance against reproductive germline editing 5. Remarkably, the NAS statement after an international summit on gene editing in 2017 shows an important shift as it changes from “not allowed as long as the risks have not been clarified” to “allowed if the risks can be assessed more reliably”. This could be interpreted as indicating that the US academies are no longer focused on a partially fundamental, partially risk‐related rejection of germline editing, but on a basic acceptance guided by individual formal and material criteria: from forbidden until criteria are met, to permitted if criteria are met—even though the criteria have not yet been agreed upon. From this perspective, the ethical rationale for editing the human genome did not change with He's experiment, but at least a year before. Against this background, it makes sense that He himself published five ethical core principles just a few days before he introduced Lulu and Nana to the world.

There is the additional issue of maintaining a distinction between therapeutic and non‐therapeutic or enhancement applications. Case in point, the deletion of the *CCR5* gene that He and his team carried out to eliminate susceptibility to the HIV virus is in a grey zone between therapy and enhancement given that transmission of the HIV virus from father to child is preventable by other means, e.g. sperm washing. Leaving aside the question of whether the edit will be effective in the intended manner.

## CRISPR and common ground

The second conceptual problem is the specific conditions under which to use emerging biotechnologies such as CRISPR. In perhaps their simplest definition, emerging biotechnologies are applications of expert knowledge to achieve practical goals. But there are at least two problems. Firstly, it implies that the end goals of a technological invention could be known in advance and that risks can be addressed accordingly 6. However, CRISPR itself is a good example that risks and difficulties, which were initially assumed to be under control, can become problematic. Second, a major lesson from the past two decades of biotechnological invention is that it is impossible to predict not only if the aims of a project will be achieved, but also if the potential applications will be societally recognized as valuable and the associated risks regarded as acceptable.

While the birth of gene‐edited children may be a rogue action, it reveals that there is insufficient oversight of the use of CRISPR or other gene‐editing technologies…

Dealing with this inherent unpredictability requires research and regulatory institutions that create stable and reliable environments for research to proceed. In this sense, institutions can be described as trusted gatekeepers: not only by ensuring that technologies are developed in an ethically and socially acceptable manner, but also by helping to settle how much and which scientific evidence is required to proceed with applications. Within this line, the crucial point surrounding He's experiment is not primarily whether He has committed misconduct, but rather if the institutional frameworks are still able—and thereby trustworthy—to provide clear guidance and norms. In order to develop scientific regimes that are able to deal with the inherent vagueness of gene‐editing biotechnologies, we have to find a common ground on the basis of which it would be possible to define the aims and the conditions under which these should be pursued.

… the ethical rationale for editing the human genome did not change with He's experiment, but at least a year before.

The public consultations on gene editing and their results are interesting and informative in this light 7, 8. Whenever there is a promise of therapeutic benefit, attitudes tend to be positive. Many interpretations of this public attitude, including He's, seem to stop there, but another trend is equally important. For example, the 2016 Pew Center reports conclude that “when it comes to using particular cutting‐edge technologies to potentially augment human abilities – such as allowing parents to edit their baby's genes for a lifetime of much reduced disease, people's concern rises”. Moreover, “[s]ome 81% of adults say gene editing that would give babies a much reduced risk of serious diseases over their lifetime would cause either a great deal of change for society (46%) or some change (35%)” 9.

## The larger picture

Too often when we speak of “ethics” in biotechnology, we think about guidelines and frameworks that regulate scientific practices. This is not wrong, but it risks missing the forest for the trees. The purpose of ethical rules in social life is, in large part, to mitigate vulnerability caused by, among other things, disease and physical suffering. Shared vulnerability can be a significant motivation for solidarity and risk‐sharing. When scientific innovation creates the possibility that some people will be able to use it to shift their level of vulnerability *vis‐à‐vis* the broader population, it raises concerns about inequality that are clearly spelled out in public consultations. It would be foolhardy and irresponsible to claim that the deletion of the *CCR5* gene undermines moral and social equality, but it would be equally remiss if we ignored the link between advances in biotechnology and broader concerns about inequality and the strength of the social fabric. Indeed, a correlation between certain types of biotech innovation and fears of an erosion of risk‐sharing or rising inequality feeds simmering suspicions about the model in which scientific research and innovation are carried out, namely the intertwining of academic research, industry, and government. In other words, the consensus on gene editing does not represent a common ground, but rather a lingering sense of insecurity.

The purpose of ethical rules in social life is, in large part, to mitigate vulnerability caused by, among other things, disease and physical suffering…

Subsequently, two questions need to be asked: “Is it safe?” and “Who benefits?” The conundrum is that trust in the response to the first question is closely linked to the response to the second. It is not just the ethical common ground that is unsettled, it is the scientific evidence itself. This is because the meaning of “safe” extends tacitly or explicitly to the potential impact on the social fabric, not just on individual patients. In this regard, it is worthwhile to remember that the stability of public knowledge—what is accepted as fact about the world—is itself politically conditioned by trust and security. Thus, responses to public ethical consultation about gene‐editing or other emerging technologies cannot be neatly distinguished from the ongoing political and economic crisis that has gripped Europe and North America since the 2008 financial crisis, which expresses itself in governments and populations that doubt “scientific facts” when it comes to issues as diverse as climate change and vaccinations. What the global scientific and policy communities need to understand is that what is accepted as scientific fact, and the common‐sense ethical evaluation of what is acceptable and unacceptable application of biotechnology cannot be separated from a broader context of institutional and political trust, confidence, and security.

… the meaning of “safe” extends tacitly or explicitly to the potential impact on the social fabric, not just on individual patients.

Openness and transparency are laudable goals for science and the ethical debate about the application of emerging technologies. But this openness does not exist in a cultural bubble of settled scientific results and evidence. If ethical and regulatory debates about CRISPR or other technologies are going to have broader societal traction and impact, scientists, ethicists, and policymakers need to realize that “the facts” of science are not settled, and that the idea of settlement is closely tied up with a broader social and political environment. Even in the “post‐truth” era, we cannot give in to a distaste for the rough and tumble of democracy as the philosopher and aristocrat Alexis de Tocqueville expressed: “I accept the intellectual rationale for democratic institutions, but I am instinctively an aristocrat, in the sense that I condemn and fear the crowd. I dearly love liberty and respect for rights, but not democracy”^10^. Scientific research is too deeply tied up with society, its impacts too wide, and the public investments too great to ignore democratic legitimation. Until the broader social and political ground has stabilized, it is unlikely that we will be able to find a common ground for the ethical debate on gene‐editing or other emerging biotechnologies.

## Conflict of interest

The authors declare that they have no conflict of interest.
